# Supplementary material for: Chromothripsis during telomere crisis is independent of NHEJ, and consistent with a replicative origin
Source: Genome Res. 2019 May;29(5):737–49. doi: 10.1101/gr.240705.118 (PMC6499312; doi:10.1101/gr.240705.118)
Supplement: Supplemental Material [file supp_gr.240705.118_Supplemental_file_1.zip › contigs/annotated_contigs/DB115/contig.4.DB115_length_1368_mean_cov_12.8961988304.docx]

**DB115_length_1368_mean_cov_12.8961988304**

```
TTGTATACTGAGGCACACTGTTTCTAGAATGAAATACAGAGGGACAGATTACAGCCTGCAAAATAACAGGAACCAGATAGAAATATGAC
 >chr18:55476389-55476701 - E=2e-175
CTTCAGAAGCCCTTAACATAGTGGTGGGTTGTGATAAATGAGATATTTCTGGAAACCAATTTGGTAAATGCATAAACATCCAAGAATAG

CAAGTGATTAAAGAAATCATCAGCAGGAGAAAATACTATGCAACTATTGAGAATTATGACCATGCCAACCATTCATTCATTCTATAAGA

ATTAATAAGCACCTGGAATGCTCTATTTAATGCTGTA|TGCCAAAA|TCATTGAGACACATTTAGTTACTTGATGTTTAATATTTCTTT
                                      >chr18:55475000-55475320 - E=3e-180
TGCAATCTAATTTCTTATTTTAAAATGTTAGTCAGAAAAAAAGAAGTCATTAAGTAGTAAAGGAAGTGGAAAGCCTTGTAACTTAGAAG

GAATGTTGGAAAACTCATTGTGCCAGAGAAAATAGAATATATCATCTGTATTTGTTGTTTTAAAAGCCATTTCGAAGGATGTGGGGCAG

ATATTTGAAAATTCTAATGTCACTTATTGCTAGGACAGATTCACATAAGAGGTGTCTTATCAGATTTAAAATTCTGGATCTGGTCTGGA

T|AA|CATCAAGTAACTAAATGTGTCTCAATGATTTTGGCAATCACACAGACTTAACATGCATAAAATGTTACAGGAGGAATTATTCAT
  >chr18:55475282-55475821 + E=4e-301
GTTATTAAAATTCACTTCCCTTTCTTTTGTATTCATAAGCATGCAAATGTGTAGGATTGTCTACCATTCTTTTCTACAAGGAAGAGATT

TTAATAAACCACTTACTGTATTTCTCTATGGAAATGTAATAAACATTACCTGATGTTGATAATGTAACACCAGTTAAACTTAATGAGTA

AAATTAATACAGACAGTTCATGAATAATTTTCCAAAATAACTTGCGATTGTTGTTAGTATATGTCGCCATGTGTTCCTGTAGTTGTAGG

AAGTTTGTATGTATTATCTTAGGCTCTGTTCTCATCATTTTCCATTGTGTCATGAAAACACATTTACCTTTTGGACTTTATCCCTCTTC

CTTCCTGACCTGCACCCTAAATGTGGCTAATTCCAGGTTGCTGTGAATAGAAGTTTACTAGATTTTTTTTTTGAGACAGTCTCACTCTG

T|C|ACCCCAAAATTTGGCATGGTTCTTTCAGAAAGGACAAGGTCGGTTTCCAGGTGAGCTAATTCCTTCATCAGCAACACCAGCTGAT
  >chr18:55474050-55474264 - E=1e-116
GCACTCTGAGAACCTCCTTCCTAAGATGAGGAAGACTTACATAGGGGAAGTGTTCTAGGATAAGGTTGTGGAGTTGTGTGGGTGTGGGT

GTTTTGTTTTGGTTTTGTTTTTTTTTTTAATTTTTTTTT
```
